# A case report of a boy suffering from type 1 diabetes mellitus and familial Mediterranean fever

**DOI:** 10.1186/s13052-021-01077-6

**Published:** 2021-06-02

**Authors:** Maria Francesca Gicchino, Dario Iafusco, Angela Zanfardino, Emanuele Miraglia del Giudice, Alma Nunzia Olivieri

**Affiliations:** Department of Woman, Child and General and Specialized Surgery, University of the Study of Campania “Luigi Vanvitelli”, Naples, Italy

**Keywords:** Recurrent fever, Abdominal pain, Arthritis, Diabetes mellitus, Colchicine, Anti interleukin 1 drugs

## Abstract

**Background:**

Type 1 diabetes mellitus could be associated with other autoimmune diseases, such as autoimmune thyroid disease, celiac disease, but the association with Familial Mediterranean Fever is rare, we describe a case of a boy with type 1 Diabetes Mellitus associated with Familial Mediterranean Fever (FMF).

**Case presentation:**

A 13 year old boy already suffering from Diabetes Mellitus type 1 since the age of 4 years, came to our attention because of periodic fever associated with abdominal pain, chest pain and arthralgia. The fever appeared every 15–30 days with peaks that reached 40 °C and lasted 24–48 h. Laboratory investigation, were normal between febrile episodes, but during the attacks revealed an increase in inflammatory markers. Suspecting Familial Mediterranean Fever molecular analysis of MEFV gene, was performed. The genetic analysis showed homozygous E148Q mutation. So Familial Mediterranean Fever was diagnosed and colchicine treatment was started with good response.

**Conclusion:**

Familial Mediterranean Fever could be associated with other autoimmune diseases such as Ankylosing Spondylitis, Rheumatoid Arthritis, Polyarteritis Nodosa, Behcet disease, Systemic Lupus, Henoch-Schönlein Purpura, and Hashimoto’s Thyroiditis. Association of type 1 Diabetes Mellitus and Familial Mediterranean Fever has been newly reported in the medical literature, this is the third association of these two diseases described in the medical literature so far.

## Background

Familial Mediterranean fever (FMF) is a monogenic autoinflammatory disease with autosomal recessive inheritance [1]. The main clinical findings of FMF are recurrent and self-limited fever attacks lasting between 12 to 72 h. Severe abdominal, articular and/or chest pain, due to inflammation of the peritoneum, synovia or pleura usually accompany fever [2]. The most important factor determining the prognosis of FMF is the development of amyloidosis, which could lead to renal failure [3]. FMF is the most common hereditary recurrent fever syndrome [1]. Approximately 150,000 people worldwide are estimated to have this condition. FMF has been report all around the world, but its prevalence is very high among certain ethnic groups such as Jewish, Turkish, Armenian and Arabs, reaching figures as high as 1/500 individuals [4].FMF result from a mutation of the Mediterranean fever (MEFV) gene, located on chromosome 16 [5] and is inherited in an autosomal recessive manner. Nearly 30% of documented FMF patients carry only one mutation, and up to 20% of patients do not have detectable mutations [6, 7]. More than 50 FMF-associated mutations in MEFV have been reported [8]. The most frequent are: M694V, V726A, M694I, and M680I located at exon 10 and E148Q located at exon 2 [1]. The MEFV gene encodes the protein pyrin, that has an important role in the inflammatory response by regulating caspase-1 activation and processing mature IL-1β [9]. The diagnosis of FMF relies mainly on clinical findings, and molecular analysis of the *MEFV* gene provides genetic confirmation [10]. There are different sets criteria for FMF diagnosis. The first set criteria was created for adults by a group of experts [11]. In 2009 Yalcikaya et al. validated a set criteria for paediatric patients [12]. Recently, the Eurofever group proposed a new set criteria for autoinflammatory recurrent fevers (these sets criteria are compared in Table 1) [13] . Colchicine is the standard treatment for FMF. However, it could be ineffective or associated with side effects in 5 to 10% of patients [13]. Interleukin-1(IL-1) inhibition could be useful in colchicine resistant FMF patients [14–16] . Canakinumab is the only biologic agent approved by the U.S. FDA for the treatment of FMF [17].

**Table 1:** Comparison of Tel-Hashomer, Yalcinkaya Ozen and Eurofer/Printo set criteria (modified from references 11,12,13). Tek-Hashomer is a writing error, it should be changed in Tel-Hashomer

Tel Hashomer Criteria	Yalcinkaya Ozen criteria	Eurofever/PRINTO clinical + genetic criteria	Eurofever/PRINTO clinical only criteria
Major Criteria 1.Recurrent febrile episodes with serosi- tis (peritonitis, synovitis or pleuritis) 2- Amyloidosis of AA type without a predisposing disease 3- Favorable response to regular colchi- cine treatment Minor Criteria 1- episodes 2- FMF in a first-degree relative 3- Erysipelas-like erythema	1- Fever (Axillary temperature of > 38 °C, 6 72 h of duration, > 3 attacks) 2- Abdominal pain (6 72 h of duration, > 3 attacks) 3- Chest pain (6 72 h of duration, > 3 attacks) 4- Arthritis (6 72 h of duration, > 3 attacks, oligoarthritis) 5- Family history of FMF	Presence of confirmatory MEFV genotype and at least one among the following 1-Duration of episodes 1–3 days 2-Arthritis 3- Chest pain 4- Abdominal pain OR Presence of not confirmatory MEFV genotype and at least two among the following 1-Duration of episodes 1–3 days 2-Arthritis 3-Chest pain 4- Abdominal pain	Presence of 1-Eastern Mediterranean ethnicity 2-Duration of episodes 1–3 days 3- Arthritis 4- Chest pain 5- Abdominal pain Absence of 1-Aphthous stomatitis 2- Urticarial rash 3- Maculopapular rash 4- Painful lymph nodes
2 major criteria or 1 major + 2 minor criteria	> 2 criteria	> 6 criteria	

Comparison of Tek-Hashomer, Yalcinkaya Ozen and Eurofer/Printo set criteria. Modified from references [11–13]

FMF could be associated with other autoimmune diseases such as ankylosing spondylitis, rheumatoid arthritis, polyarteritis nodosa, Behcet,, and Systemic Lupus, and Hashimoto’s thyroiditis [18, 19]. Association of type 1 diabetes mellitus (T1D) and FMF has been newly reported in the medical literature [20, 21]. We report a case of a boy suffering from T1D, who developed FMF. This is the third association of these two diseases described in the medical literature.

## Case report

The patient was in follow up in the Paediatric Diabetological Center of our Department because he developed T1D at the age of 4. At the age of 13 he was referred to the Paediatric Rheumatological Centre of our Department because of episodes of recurrent fever since the age of 6. Fever attacks, with temperature ranging between 39 to 40 °C, lasted 24 to 72 h and occurred every 21–30 days. These episodes where associated with arthralgia, abdomen, and chest pain.

Over the years because of fever and abdominal pain, the patient usually referred to Emergency, where he underwent to abdomen ultrasounds in order to exclude acute appendicitis. Blood tests performed during fever attacks showed increase in inflammatory parameters, erythrocyte sedimentation rate (ERS) and C-reactive protein (CRP).

Fever attacks were treated with antipyretic drugs and in some occasion with antibiotic. Between attacks patient was well and blood tests were normal. The patient came to our attention during a febrile attack. Physical examination revealed: fever (body temperature up to 40 °C), abdominal and chest pain, arthritis of the right ankle. Blood tests revealed an increase in white cells (17.000/mm^3^, normal value < 10.000/mm^3^), ERS (60 mm/ h), CRP (3.7 mg/dl) serum amyloid A (SAA,200 mg/dL). Blood tests were unremarkable for: viral serology, liver and kidney function, serum immunoglobulins,coeliac screening, thyroid hormone, antinuclear antibodies, extractable nuclear antigens, anti neutrophil Cytoplasmic antibodies, rheumatoid factor, anti-double stranded DNA, serum antistreptolysin O titre and complement levels. Throat swab was negative and so were the urinalysis, and the abdomen ultrasound. Chest ultrasound showed pleural effusion. Given his personal history, clinical and laboratory findings FMF was suspected, so colchicine therapy (1 mg/day) was prescribed and genetic investigation was performed. The molecular analysis of MEFV gene, showed homozygous E148Q mutation. Colchicine determined the immediate disappearance of the symptoms and normalization of inflammatory parameters. Colchicine was well tolerated.

After 1 year a renal biopsy was performed because of onset of persistent microalbuminuria. On biopsy Congo red staining was negative, so amyloidosis was excluded, but a slight and irregular thickening of the lamina densa of some glomerular capillaries was detected, so diabetic nephropathy was diagnosed and Ramipril treatment (2, 5 mg/day) was prescribed. Regarding FMF, after 18 months without symptoms, the patient presented again fever and abdominal pain, associated with an increase in inflammatory parameters, so colchicine was increased to 1.5 mg/day. On his recent follow up visit, at 17 years of age, the patient was in good general conditions. Daily insulin requirement was 0,8 U/kg/day. Patient did not refer fever or abdominal pain, and urynalysis did not reveal microalbuminuria. As he did not refer any side effects, both colchicine (1,5 mg/day) and ramipril (2,5 mg/day) were confirmed.

## Discussion and conclusion

FMF is an autoinflammatory disease with autosomal recessive inheritance. Our patient presented homozygous E148Q mutation. E148Q is the most frequent variant among carriers, its pathogenic role is uncertain [22]. In a recent analysis Topaloglu et al. demonstrated that patients homozygous for E148Q displayed typical FMF phenotype and half of these patients had moderate/severe disease before colchicine treatment [22].

Aydin et al. demonstrated that E148Q mutation is associated with a milder disease course, despite patients may have similar clinical findings and well response to colchicine therapy, when compared to patients with other mutations [23] . In our case, the patient presented recurrent fever episodes associated to abdominal, chest pain, and arthralgia and presented a good response to colchicine treatment.

It has been reported that E148Q mutation could induce a nonamyloidosis renal involvement. In particular Eroglu et al. described a case of mesangial proliferative glomerulonephritis in a woman affected by FMF with an heterozygous E148Q mutation [24]. Ardalan et al. reported a case of an acute glomerulonephritis with proteinuria in a patient affected from FMF with an heterozygous E148Q mutation [25]. Our patient presented persistent microalbuminuria 1 year later FMF diagnosis, so a renal biopsy was performed that revealed slight and irregular thickening of the lamina densa of some glomerular capillaries. This histopathological finding was compatible with diabetic nephropathy, so treatment with Ramipril was started [26].

Pyrin, the protein product of MEFV, is a 781-aminoacid protein expressed in serosal and synovial fibroblasts, granulocytes, and cytokine-activated monocytes.

The role of pyrin in IL-1 activation is controversial [8], Campbell et al. supposed that pyrin suppresses the activation of pro-caspase-1, by competing for ASC (apoptosis-associated speck-like protein containing a caspase recruitment domain), and therefore pyrin interferes with NALP3 inflammasome activation [8]. Chae et al demonstrated that Pyrin containing FMF-associated mutations has less of an inhibitory effect on the inflammasome, leading to upregulated synthesis of IL-1β [9].

T1D is a T-cell–mediated autoimmune disease characterized by the destruction of pancreatic beta cells in genetically predisposed individuals [27].

As in other autoimmune conditions, both innate and adaptive immunity play a role in disease pathogenesis [28, 29].

Kumar et al. demonstrated that T-helper type 17 (Th17) cells, have a pivotal role in T1D pathogenesis [30]. Proinflammatory cytokine, in particular interleukin 1 (IL-1) and 6 (IL-6), are involved in differentiation of T-cells in Th17 [30]. It has been demonstrated that TNFα, IL-1 and IL-6 are increased in diabetic subjects compared to control subjects at onset of clinical disease. These cytokine inducing differentiation of T-cells in Th17 are involved in T1D pathogenesis [31, 32].

Recent studies have demonstrated NLRP3 inflammosome and IL-1 iper-production could play an important role in the development of T1D in mice [33]. The coexistence of FMF and type T1D is a rare finding. In 2006 Atabek et al. described a case of a 9 years old girl affected from T1D who developed FMF 11 months later diabetes onset [20]. In 2009 Baş et al. reported a second patient with type T1D associated with FMF who also had autoimmune thyroid disease (ATD), celiac disease (CD) [21].

According to the recent progress in the understanding of T1D pathogenesis, in particular regarding the increase in serum levels of IL1 and IL6 at the onset of diabetes, we can suppose that the higher production of IL1 in FMF could be involved in development of T1D. Further studies or case series are needed to demonstrate this possible association.

Here we report the third association of FMF and T1D. FMF should be kept in mind in the differential diagnosis of disorders associated with T1D in the presence of recurrent and limited attacks of fever, associated with abdominal or chest pain or arthritis. The emerging role of the inflammatory cytokine (TNFα, IL-1 and IL-6)in the development of T1D adds a further dimension to our understanding of the multifactorial nature of the immunopathology that leads to the development of T1D but also opens a new area of research for potential therapy.

## Data Availability

Not applicable.

## Abbreviations

FMF: Familial Mediterranean Fever
IL-1: Interleukine 1
IL-6: Interleukine 6
T1D: Type 1 diabetes mellitus
ERS: Erythrocyte sedimentation rate
CRP: C-reactive protein
SAA: Serum amyloid A
ASC: Apoptosis-associated speck-like protein containing a caspase recruitment domain
TNFα: Tumor necrosis factor alfa
Th-17: T-helper type 17
ATD: Autoimmune thyroid disease
CD: Celiac disease
